# Variability in Medullary Thyroid Carcinoma in *RET* L790F Carriers: A Case Comparison Study of Index Patients

**DOI:** 10.3389/fendo.2020.00251

**Published:** 2020-04-28

**Authors:** Jes Sloth Mathiesen, Søren Grønlund Nielsen, Åse Krogh Rasmussen, Katalin Kiss, Karin Wadt, Anne Pernille Hermann, Morten Frost Nielsen, Stine Rosenkilde Larsen, Klaus Brusgaard, Anja Lisbeth Frederiksen, Christian Godballe, Maria Rossing

**Affiliations:** ^1^Department of ORL Head and Neck Surgery and Audiology, Odense University Hospital, Odense, Denmark; ^2^Department of Clinical Research, University of Southern Denmark, Odense, Denmark; ^3^Department of Medical Endocrinology, Copenhagen University Hospital, Copenhagen, Denmark; ^4^Department of Pathology, Copenhagen University Hospital, Copenhagen, Denmark; ^5^Department of Clinical Genetics, Copenhagen University Hospital, Copenhagen, Denmark; ^6^Department of Endocrinology, Odense University Hospital, Odense, Denmark; ^7^Department of Pathology, Odense University Hospital, Odense, Denmark; ^8^Department of Clinical Genetics, Odense University Hospital, Odense, Denmark; ^9^Department of Clinical Genetics, Aalborg University Hospital, Aalborg, Denmark; ^10^Center for Genomic Medicine, Copenhagen University Hospital, Copenhagen, Denmark

**Keywords:** multiple endocrine neoplasia type 2, medullary thyroid carcinoma, *RE*arranged during *T*ransfection, L790F, variability, gene variants, *FLT3* R387Q

## Abstract

**Background:** Previous studies have suggested that the variability in age of onset and aggressiveness of medullary thyroid carcinoma (MTC) in patients with multiple endocrine neoplasia type 2A (MEN 2A) carrying the same *RE*arranged during *T*ransfection (*RET*) mutation may be caused by additional *RET* germline variants or somatic variants.

**Methods:** This study was a retrospective case comparison study of all MEN 2A index patients (*n* = 2) with the *RET* L790F germline mutation in Denmark. Whole blood and MTC tissue were analyzed for *RET* germline variants and other somatic variants (>500), respectively.

**Results:** Patient 1 presented with MTC (T1aN1bM0) at age 14 years, while patient 2 presented with MTC (T1bN0M0) at age 70 years. No germline *RET* germline variants nor other variants were found to explain this MTC variability.

**Conclusions:** We could not confirm the previously reported finding of a somatic *RET* variant as likely responsible for the early onset and aggressiveness of MTC in a *RET* germline mutation carrier. Also, we found no *RET* germline variants that could explain the MTC variability among our index patients. We did, however, identify a somatic *FLT3* R387Q variant with an unknown potential as genetic modifier. Further large-scale studies are needed to investigate genetic modifiers in *RET* L790F carriers.

## Introduction

Multiple endocrine neoplasia (MEN) 2 is an autosomal dominant inherited cancer syndrome subdivided into MEN 2A and MEN 2B (1, 2). With a point prevalence of 13–24 and 1–2 per million, MEN 2A and MEN 2B are rare (3–6). The syndromes, however, are accompanied by major implications as virtually all patients develop medullary thyroid carcinoma (MTC) while variable proportions develop pheochromocytoma, primary hyperparathyroidism, cutaneous lichen amyloidosis, Hirschsprung disease, ganglioneuromatosis of the aerodigestive tract, facial, ophthalmologic, and skeletal abnormalities (1).

MEN 2A and 2B are caused by germline mutations of the *RE*arranged during *T*ransfection (*RET*) proto-oncogene (7–11). Despite the recognition of strong genotype-phenotype correlations (12, 13), several studies have observed a substantial inter- and intra-familial variability in the age of onset and aggressiveness of MTC among carriers of the same mutation (14–21). Some authors have suggested that certain *RET* germline variants (IVS1-126G>T, IVS8+82A>G; 85-86 insC, G691S, L769L, S836S, S904S) in combination with a *RET* germline mutation may modify the age of onset and aggressiveness of MTC in MEN 2 patients (22–26). The issue, however, is controversial (25–30). Others have suggested that the variability may be caused by a somatic variant acting as the “second hit” in Knudson's two hit model (16, 31, 32). To our knowledge, no such studies exist for MEN 2A patients carrying the *RET* L790F germline mutation.

Consequently, we conducted the first study of *RET* germline variants and somatic variants in L790F carriers, aiming to explain the MTC variability among index patients in Denmark.

## Methods

### Study Design and Participants

This investigation is a retrospective case study of all MEN 2A index patients (*n* = 2) with the *RET* L790F germline mutation in Denmark.

### Data Sources

Patients were identified from the extended nationwide Danish *RET* cohort 1994–2017 (33, 34), and data were drawn from the Danish MEN 2A cohort 1901–2014, the Danish MTC cohort 1997–2014, and medical records (4, 35).

### Genetic Testing

Testing for *RET* germline variants were performed using Next Generation Sequencing as previously described (33). Formalin-fixed paraffin-embedded tumoir tissue from MTC primary tumor and metastases were tested for somatic *RET* variants as reported earlier (36). Testing for somatic variants in oncogenes was done by targeted exome sequencing; In short, genomic DNA (200 ng) was fragmented to 300 bp adaptor ligation using KAPA HTP Library Preparation Kit (Roche) and enrichment of exome using SureSelectXT Clinical Research Exome kit (Agilent). Paired-end sequencing (2 × 100 or 2 × 150 bp) was performed to gain an average coverage of 50–100 × using the NextSeq500 platforms (Illumina). Raw sequencing data were processed using CASAVA-1.8.2 and reads were aligned to the human reference genome (hg19/GRCh37) using CLC Biomedical Genomics Workbench (Qiagen). Variant calling >5% frequency and filtering for 523 cancer relevant genes, including *RET, H-RAS, K-RAS*, and *N-RAS* using Ingenuity Variant Analysis (Qiagen). For this study, “mutation” denotes a disease-causing (pathogenic) sequence change, while “variant” denotes any sequence change, whether pathogenic or not.

The investigation was approved by the Danish Health Authority (3-3013-395/3) and the Danish Data Protection Agency (18/17801). The Regional Committees on Health Research Ethics for Southern Denmark found further review was not liable to notification. Informed consent was obtained from the cases for the publication of any potentially identifiable images or data included in this article. The data that support the findings of this study are available from the corresponding author upon request.

## Results

### Patient 1

A 14-year-old female of Lebanese origin, presented with B-symptoms and multiple enlarged lymph nodes on the right side of the neck. Fine-needle aspiration suggested plasma cells or cells with thyroid or paraganglioma origin. A lymph node biopsy was taken and histology showed MTC metastasis. After this, basal calcitonin was measured to 18.3 μg/L (<0.1 μg/L). The patient underwent a total thyroidectomy and right-sided neck dissection. Histology displayed multifocal MTC (largest tumor 7 mm) with metastases to 17 of 20 removed lymph nodes and a normal parathyroid gland. The final TNM-stage was T1aN1bM0. Two months postoperatively basal calcitonin was 19.0 μg/L (<0.1 μg/L) and adjuvant radiotherapy was given. *RET* testing revealed the L790F (c.2370G>T) germline mutation. There was no family history of MEN 2A. The father was tested negative for *RET* mutations, while the mother was positive for the L790F mutation. The latter, however, declined further work up. Fourteen years later basal calcitonin had risen to 59,280 ng/L (<7.3 ng/L). Distant metastases were cytologically verified in mediastinal lymph nodes and suspected in the neck, lungs, mamma, liver, and in proximity to the transverse colon. The patient was initiated on Vandetanib, a tyrosine kinase inhibitor. Thirty months after initiation, the patient had basal calcitonin of 80 ng/L and apart from the suspected liver metastasis that was assessed without change there were no radiological evidence of metastases on CT scan. Neither pheochromocytoma nor primary hyperparathyroidism has been diagnosed during the 16 years of follow-up. Table 1 shows clinical characteristics and results of genetic analyses for patient 1.

**Table 1:** Clinical and genetic characteristics of two index patients with the *RET* L790F germline mutation.

		**Patient 1**	**Patient 2**
Sex		Female	Female
Ethnic origin		Lebanese	Danish
MTC at diagnosis			
Age, years		14	70
Largest tumor size, mm		7	15
Cervical lymph nodes metastasized/removed		17/20	0/1
*RET* germline variants			
IVS1-126G>T	(rs2565206)	–	–
A45A	(rs1800858)	+	+
c.337+9G>A	(rs2435351)	+	+
IVS4+48A>G	(rs2435352)	+	+
IVS8+82A>G; 85–86 insC	(rs3026750; rs3482797)	–	–
A432A	(rs1800860)	–	+
G691S	(rs1799939)	–	+
IVS12+47C>T	(rs760466)	+	–
L769L	(rs1800861)	+	+
L790F	(rs75030001)	+	+
S836S	(rs1800862)	–	–
S904S	(rs1800863)	–	+
IVS19+47C>T	(rs2075912)	+	+
Somatic variants			
*RET*		–	–
*N-RAS, K-RAS*, or *H*-*RAS*		–	–
*FLT3* (R387Q)	(rs751562024)	+	–
Other genes analyzed (>500)^a^		–	–

*MTC, medullary thyroid carcinoma; RET, REarranged during Transfection*.

a *List of genes analyzed can be found in Appendix 1*.

### Patient 2

A 70-year-old female, presented with a right-sided thyroid tumor, but was otherwise asymptomatic. Fine-needle aspiration showed follicular neoplasm of unknown malignant potential. The medical history was unremarkable apart from a right-sided ductal carcinoma in situ treated with lumpectomy and radiotherapy 18 months earlier. The patient underwent a left-sided hemithyroidectomy without prior measurement of serum calcitonin, as MTC was unsuspected. Histology revealed multifocal MTC with the largest tumor size of 15 mm. At this point, basal serum calcitonin was 28 ng/L (<7.3 ng/L) and family history revealed that the mother and aunt had undergone thyroid operations for unknown causes. The patient underwent right-sided completion thyroidectomy. Histology showed multifocal MTC with several tumors measuring 1–3 mm. Also, a removed lymph node was without metastasis. The final TNM-stage was T1bN0M0. A *RET* test identified the L790F (c.2370G>T) germline mutation. Subsequently, normal tissue from the deceased mother was also tested positive for the L790F mutation. Basal serum calcitonin was immeasurable and no biochemical signs of pheochromocytoma or primary hyperparathyroidism have been present after 1 year of follow-up. Clinical characteristics and results of genetic analyses for patient 2 can be seen in Table 1.

### Genetic Characteristics

The only *RET* germline variant and the only somatic variant exclusively identified in patient 1 were IVS12+47C>T and *FLT3* R387Q (c.1160G>A), respectively. The *RET* variants exclusively found in patient 2 were A432A, G691S, and S904S.

## Discussion

Despite thorough investigations, this study found no *RET* germline variants or other somatic variants that could convincingly explain the substantial variability in MTC phenotype seen in our two L790F index patients.

Patient 1 presented with MTC and lymph node metastases at 14 years old, several years earlier than the reported median age (57 years) for MTC presentation in L790F index patients (37). The only *RET* germline variant exclusively identified in this patient was IVS12+47C>T. While other *RET* intron variants (IVS1-126G>T, IVS8+82A>G; 85-86 insC) have been associated to earlier age at MTC onset and the presence of lymph node metastases at diagnosis (26), no similar association has been reported for the *RET* IVS12+47C>T variant. The variant is classified as benign in the ClinVar database according to the criteria from the American College of Medical Genetics and Genomics (38, 39). To the best of our knowledge, no functional studies of this variant exist.

**Figure 1:**
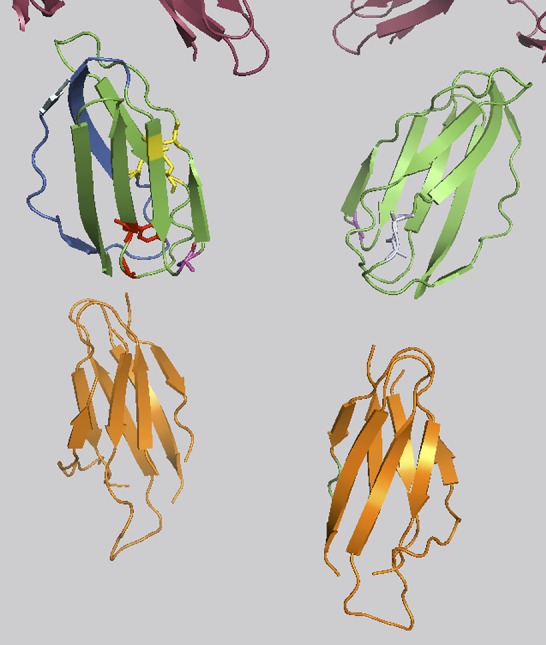
FLT3: FL interaction. D4 interface taking part in FL binding shown in marine blue. Conserved disulfid bridges of the FLT3 Homotype interacting D4 domains in yellow. The highly conserved Tyrosin of the EF-Loop is shown in red. D5 domains in orange. The variation R387 in magenta. Glutamines opposing R387 involved in homodimerisation shown in light grey. The upper part of the figure (in raspberry) represents part of the D1, D2 and D3 domains.

The only somatic variant exclusively found in patient 1 was *FLT3* R387Q (c.1160G>A). Fms-like tyrosine kinase 3 ligand (FL) is the cytokine ligand of FLT3, which is considered an important growth and differentiation factor. FLT3 is commonly found in hematopoietic malignancies. In most cases, this is due to activating mutations in the FLT3 gene but a significant number of leukemias are also characterized by a higher than normal expression level of un-mutated, wild-type FLT3, thus emphasizing the importance of FLT3 signaling perturbations in malignant transformation. FL interacts with FLT3 at the EF-loop of the homodimeric FLT3. R387 is situated in the D4 domain of FLT3 interacting with FL (Figure 1) and is part of the interface of D4 taking part in homodimerization. R387 is part of the FG-loop situated next to the highly conserved EF-loop constituting the tyrosine corner. In the majority of other tyrosine kinase receptors the homodimerization is achieved by hydrogen bonds between argenines and glutamines of the FG-Loop. FLT3 show sequence dissimilarity in this particular region of the D4 domain region to other tyrosine kinase receptors. It can be hypothesized that R387Q could be part of a hydrogen binding motif with the opposing Glutamine 365 or 366 in the FLT3 partner. Thus, we cannot exclude a possible genetic modifying role for the R387Q variant.

Patient 2 presented relatively late with an intrathyroidal MTC at 70 years old. Variants exclusively found in this patient were A432A, G691S, and S904S. Of these variants, the G691S and S904S variant and their association to MTC have been investigated in several MEN 2A studies (22, 23, 25–27, 29). A study of 104 MEN 2A patients from Spain suggested that G691S and S904S could be related to early appearance of symptoms in an overall MEN 2A group and in a group with codon 634 mutations only (22). Similarly, a recent Italian study observed an earlier age at MTC diagnosis in patients carrying the G691S variant and the S891A germline mutation compared to those only carrying the S891A mutation (23). However, in the present study, we could not confirm a genetic modifier effect of the G691S and S904S variant when comparing our two L790F index patients. This is in accordance with other investigations that studied the G691S and S904S variant in MEN 2A patients carrying mutations of codon 533, 618, and 634 (25–27, 29).

The last variant exclusively found in patient 2 was A432A. An international collaborative study of 384 MEN 2A patients from four different European populations suggested a protective effect of the A432A variant with a 50% decreased risk of developing pheochromocytoma and/or primary hyperparathyroidism (*P* = 0.03) (27). Such an effect cannot be ruled out for patient 2, as she has not presented with other MEN 2A manifestations despite a relatively advanced age at MTC diagnosis. However, the penetrance of pheochromocytoma and primary hyperparathyroidism in L790F carriers has been reported as exceedingly low (20). Although pheochromocytoma often is reported in carriers of codon 634 mutations (40–45), there have also been reports of this manifestation in L790F carriers (20, 46–48). Opposed to the phenotype of our two patients, having only MTC and no pheochromocytoma, a 44-year-old L790F carrier has been reported to present with bilateral pheochromocytoma and no evidence of MTC. The report underlines the need for MEN 2 screening in all patients with pheochromocytoma and the need for lifelong screening for pheochromocytoma in all MEN 2 patients (46, 49).

We found no somatic variants in *RET, H-RAS, K-RAS*, and *N-RAS* in neither of our MEN 2A index patients. This is consistent with some studies (50–53), but in contrast to others (54, 55). None of these studies correlated the absence or presence of somatic variants to the age at diagnosis and aggressiveness of MTC. This was, however, done in a French-Italian collaborative study of V804L carriers (16). Despite V804L carriers usually displaying a late onset and an indolent course of C-cell disease, the authors reported of an index patient, who presented with a thyroid tumor (25 mm), clinical lymph node involvement, and elevated basal calcitonin (1,750 pg/ml) at 12 years of age. A somatic M918T mutation was detected in the patient's tumor. The authors hypothesized that the two mutations were likely responsible for both the early clinical onset and the aggressiveness of the tumor (16). The lack of somatic *RET* variants in both our index patients suggests that a similar hypothesis may not be valid for L790F carriers. However, future large-scale studies are needed to adequately address the question. Such studies are also needed to provide more information of useful genotype-phenotype correlations for better prognostication in L790 carries. The present investigation is limited by the lack of functional studies although they can be misleading from time to other (36, 56).

As patient 1 was of Lebanese origin (immigrated to Denmark at four years of age) and patient 2 of Danish origin, influence of ethnic, and geographical factors cannot be completely ruled out. Even though other studies have found geographical differences in pheochromocytoma of MEN 2A and survival of MEN 2B patients, the issue remains hypothetical (57, 58).

## Conclusions

We could not confirm the previously reported finding of a somatic *RET* variant as likely responsible for the early onset and aggressiveness of MTC in a *RET* germline mutation carrier. Also, we found no *RET* germline variants that could explain the MTC variability among our index patients. We did, however, identify a somatic *FLT3* R387Q variant with an unknown potential as genetic modifier. Further large-scale studies are needed to investigate genetic modifiers in *RET* L790F carriers.

## Data Availability Statement

Due to anonymity of the two cases reported in the article, we would prefer not to deposit the sequencing data in public community-supported repository. However, the data are available upon reasonable request to the authors.

## Ethics Statement

Written informed consent was obtained from the cases for the publication of any potentially identifiable images or data included in this article.

## Author Contributions

JM conceived the study, collected data, and drafted the manuscript. MR contributed with genetic analyses, data collection, and manuscript drafting. KK and SL contributed with collection of tumor tissue and manuscript drafting. KB contributed with interpretation of variants and manuscript drafting. SN, ÅR, KW, AH, MN, AF, and CG contributed with data collection and manuscript drafting.

## Conflict of Interest

The authors declare that the research was conducted in the absence of any commercial or financial relationships that could be construed as a potential conflict of interest.
