# Supplementary material for: Variability in Medullary Thyroid Carcinoma in RET L790F Carriers: A Case Comparison Study of Index Patients
Source: Front Endocrinol (Lausanne). 2020 Apr 28;11:251. doi: 10.3389/fendo.2020.00251 (PMC7198720; doi:10.3389/fendo.2020.00251)
Supplement: Supplementary file 1 [file Data_Sheet_1.docx]

**Appendix 1.** Genes included in the TruSight Oncology 500 panel.

| **DNA content** | | | | | | | | | | |
| --- | --- | --- | --- | --- | --- | --- | --- | --- | --- | --- |
| *ABL1* | *BRD4* | *CUX1* | *FAM175A* | *GATA6* | *IGF1* | *MAP3K13* | *NOTCH4* | *POLE* | *RPTOR* | *TAF1* |
| *ABL2* | *BRIP1* | *CXCR4* | *FAM46C* | *GEN1* | *IGF1R* | *MAP3K14* | *NPM1* | *PPARG* | *RUNX1* | *TBX3* |
| *ACVR1* | *BTG1* | *CYLD* | *FANCA* | *GID4* | *IGF2* | *MAP3K4* | *NRAS* | *PPM1D* | *RUNX1T1* | *TCEB1* |
| *ACVR1B* | *BTK* | *DAXX* | *FANCC* | *GLI1* | *IKBKE* | *MAPK1* | *NRG1* | *PPP2R1A* | *RYBP* | *TCF3* |
| *AKT1* | *C11orf30* | *DCUN1D1* | *FANCD2* | *GNA11* | *IKZF1* | *MAPK3* | *NSD1* | *PPP2R2A* | *SDHA* | *TCF7L2* |
| *AKT2* | *CALR* | *DDR2* | *FANCE* | *GNA13* | *IL10* | *MAX* | *NTRK1* | *PPP6C* | *SDHAF2* | *TERC* |
| *AKT3* | *CARD11* | *DDX41* | *FANCF* | *GNAQ* | *IL7R* | *MCL1* | *NTRK2* | *PRDM1* | *SDHB* | *TERT* |
| *ALK* | *CASP8* | *DHX15* | *FANCG* | *GNAS* | *INHA* | *MDC1* | *NTRK3* | *PREX2* | *SDHC* | *TET1* |
| *ALOX12B* | *CBFB* | *DICER1* | *FANCI* | *GPR124* | *INHBA* | *MDM2* | *NUP93* | *PRKAR1A* | *SDHD* | *TET2* |
| *ANKRD11* | *CBL* | *DIS3* | *FANCL* | *GPS2* | *INPP4A* | *MDM4* | *NUTM1* | *PRKCI* | *SETBP1* | *TFE3* |
| *ANKRD26* | *CCND1* | *DNAJB1* | *FAS* | *GREM1* | *INPP4B* | *MED12* | *PAK1* | *PRKDC* | *SETD2* | *TFRC* |
| *APC* | *CCND2* | *DNMT1* | *FAT1* | *GRIN2A* | *INSR* | *MEF2B* | *PAK3* | *PRSS8* | *SF3B1* | *TGFBR1* |
| *AR* | *CCND3* | *DNMT3A* | *FBXW7* | *GRM3* | *IRF2* | *MEN1* | *PAK7* | *PTCH1* | *SH2B3* | *TGFBR2* |
| *ARAF* | *CCNE1* | *DNMT3B* | *FGF1* | *GSK3B* | *IRF4* | *MET* | *PALB2* | *PTEN* | *SH2D1A* | *TMEM127* |
| *ARFRP1* | *CD274* | *DOT1L* | *FGF10* | *H3F3A* | *IRS1* | *MGA* | *PARK2* | *PTPN11* | *SHQ1* | *TMPRSS2* |
| *ARID1A* | *CD276* | *E2F3* | *FGF14* | *H3F3B* | *IRS2* | *MITF* | *PARP1* | *PTPRD* | *SLIT2* | *TNFAIP3* |
| *ARID1B* | *CD74* | *EED* | *FGF19* | *H3F3C* | *JAK1* | *MLH1* | *PAX3* | *PTPRS* | *SLX4* | *TNFRSF14* |
| *ARID2* | *CD79A* | *EGFL7* | *FGF2* | *HGF* | *JAK2* | *MLL* | *PAX5* | *PTPRT* | *SMAD2* | *TOP1* |
| *ARID5B* | *CD79B* | *EGFR* | *FGF23* | *HIST1H1C* | *JAK3* | *MLLT3* | *PAX7* | *QKI* | *SMAD3* | *TOP2A* |
| *ASXL1* | *CDC73* | *EIF1AX* | *FGF3* | *HIST1H2BD* | *JUN* | *MPL* | *PAX8* | *RAB35* | *SMAD4* | *TP53* |
| *ASXL2* | *CDH1* | *EIF4A2* | *FGF4* | *HIST1H3A* | *KAT6A* | *MRE11A* | *PBRM1* | *RAC1* | *SMARCA4* | *TP63* |
| *ATM* | *CDK12* | *EIF4E* | *FGF5* | *HIST1H3B* | *KDM5A* | *MSH2* | *PDCD1* | *RAD21* | *SMARCB1* | *TRAF2* |
| *ATR* | *CDK4* | *EML4* | *FGF6* | *HIST1H3C* | *KDM5C* | *MSH3* | *PDCD1LG2* | *RAD50* | *SMARCD1* | *TRAF7* |
| *ATRX* | *CDK6* | *EP300* | *FGF7* | *HIST1H3D* | *KDM6A* | *MSH6* | *PDGFRA* | *RAD51* | *SMC1A* | *TSC1* |
| *AURKA* | *CDK8* | *EPCAM* | *FGF8* | *HIST1H3E* | *KDR* | *MST1* | *PDGFRB* | *RAD51B* | *SMC3* | *TSC2* |
| *AURKB* | *CDKN1A* | *EPHA3* | *FGF9* | *HIST1H3F* | *KEAP1* | *MST1R* | *PDK1* | *RAD51C* | *SMO* | *TSHR* |
| *AXIN1* | *CDKN1B* | *EPHA5* | *FGFR1* | *HIST1H3G* | *KEL* | *MTOR* | *PDPK1* | *RAD51D* | *SNCAIP* | *U2AF1* |
| *AXIN2* | *CDKN2A* | *EPHA7* | *FGFR2* | *HIST1H3H* | *KIF5B* | *MUTYH* | *PGR* | *RAD52* | *SOCS1* | *VEGFA* |
| *AXL* | *CDKN2B* | *EPHB1* | *FGFR3* | *HIST1H3I* | *KIT* | *MYB* | *PHF6* | *RAD54L* | *SOX10* | *VHL* |
| *B2M* | *CDKN2C* | *ERBB2* | *FGFR4* | *HIST1H3J* | *KLF4* | *MYC* | *PHOX2B* | *RAF1* | *SOX17* | *VTCN1* |
| *BAP1* | *CEBPA* | *ERBB3* | *FH* | *HIST2H3A* | *KLHL6* | *MYCL1* | *PIK3C2B* | *RANBP2* | *SOX2* | *WISP3* |
| *BARD1* | *CENPA* | *ERBB4* | *FLCN* | *HIST2H3C* | *KMT2B* | *MYCN* | *PIK3C2G* | *RARA* | *SOX9* | *WT1* |
| *BBC3* | *CHD2* | *ERCC1* | *FLI1* | *HIST2H3D* | *KMT2C* | *MYD88* | *PIK3C3* | *RASA1* | *SPEN* | *XIAP* |
| *BCL10* | *CHD4* | *ERCC2* | *FLT1* | *HIST3H3* | *KMT2D* | *MYOD1* | *PIK3CA* | *RB1* | *SPOP* | *XPO1* |
| *BCL2* | *CHEK1* | *ERCC3* | *FLT3* | *HLA-A* | *KRAS* | *NAB2* | *PIK3CB* | *RBM10* | *SPTA1* | *XRCC2* |
| *BCL2L1* | *CHEK2* | *ERCC4* | *FLT4* | *HLA-B* | *LAMP1* | *NBN* | *PIK3CD* | *RECQL4* | *SRC* | *YAP1* |
| *BCL2L11* | *CIC* | *ERCC5* | *FOXA1* | *HLA-C* | *LATS1* | *NCOA3* | *PIK3CG* | *REL* | *SRSF2* | *YES1* |
| *BCL2L2* | *CREBBP* | *ERG* | *FOXL2* | *HNF1A* | *LATS2* | *NCOR1* | *PIK3R1* | *RET* | *STAG1* | *ZBTB2* |
| *BCL6* | *CRKL* | *ERRFI1* | *FOXO1* | *HNRNPK* | *LMO1* | *NEGR1* | *PIK3R2* | *RFWD2* | *STAG2* | *ZBTB7A* |
| *BCOR* | *CRLF2* | *ESR1* | *FOXP1* | *HOXB13* | *LRP1B* | *NF1* | *PIK3R3* | *RHEB* | *STAT3* | *ZFHX3* |
| *BCORL1* | *CSF1R* | *ETS1* | *FRS2* | *HRAS* | *LYN* | *NF2* | *PIM1* | *RHOA* | *STAT4* | *ZNF217* |
| *BCR* | *CSF3R* | *ETV1* | *FUBP1* | *HSD3B1* | *LZTR1* | *NFE2L2* | *PLCG2* | *RICTOR* | *STAT5A* | *ZNF703* |
| *BIRC3* | *CSNK1A1* | *ETV4* | *FYN* | *HSP90AA1* | *MAGI2* | *NFKBIA* | *PLK2* | *RIT1* | *STAT5B* | *ZRSR2* |
| *BLM* | *CTCF* | *ETV5* | *GABRA6* | *ICOSLG* | *MALT1* | *NKX2-1* | *PMAIP1* | *RNF43* | *STK11* | *ZRSR2* |
| *BMPR1A* | *CTLA4* | *ETV6* | *GATA1* | *ID3* | *MAP2K1* | *NKX3-1* | *PMS1* | *ROS1* | *STK40* | *ZRSR2* |
| *BRAF* | *CTNNA1* | *EWSR1* | *GATA2* | *IDH1* | *MAP2K2* | *NOTCH1* | *PMS2* | *RPS6KA4* | *SUFU* | *ZRSR2* |
| *BRCA1* | *CTNNB1* | *EZH2* | *GATA3* | *IDH2* | *MAP2K4* | *NOTCH2* | *PNRC1* | *RPS6KB1* | *SUZ12* | *ZRSR2* |
| *BRCA2* | *CUL3* | *FAM123B* | *GATA4* | *IFNGR1* | *MAP3K1* | *NOTCH3* | *POLD1* | *RPS6KB2* | *SYK* | *ZRSR2* |
| **RNA content*** | | | | | | | | | | |
| ABL1 | BCL2 | CSF1R | ESR1 | EWSR1 | FLI1 | KIF5B | MSH2 | NRG1 | PAX7 | RAF1 |
| AKT3 | BRAF | EGFR | ETS1 | FGFR1 | FLT1 | KIT | MYC | NTRK1 | PDGFRA | RET |
| ALK | BRCA1 | EML4 | ETV1 | FGFR2 | FLT3 | MET | NOTCH1 | NTRK2 | PDGFRB | ROS1 |
| AR | BRCA2 | ERBB2 | ETV4 | FGFR3 | JAK2 | MLL | NOTCH2 | NTRK3 | PIK3CA | RPS6KB1 |
| AXL | CDK4 | ERG | ETV5 | FGFR4 | KDR | MLLT3 | NOTCH3 | PAX3 | PPARG | TMPRSS2 |

*The products to evaluate DNA and RNA variants consist of the TruSight Oncology 500 DNA panel and the TruSight Tumor 170 RNA panel.
